# Risk Factors for Tumor Positive Resection Margins After Neoadjuvant Chemoradiotherapy for Esophageal Cancer: Results From the Dutch Upper GI Cancer Audit: A Nationwide Population-Based Study

**DOI:** 10.1097/SLA.0000000000005112

**Published:** 2023-01-10

**Authors:** Ingmar L. Defize, Lucas Goense, Alicia S. Borggreve, Stella Mook, Gert J. Meijer, Jelle P. Ruurda, Richard van Hillegersberg

**Affiliations:** * Department of Surgery, University Medical Center Utrecht, Utrecht, The Netherlands; † Department of Radiation Oncology, University Medical Center Utrecht, Utrecht, The Netherlands

**Keywords:** esophageal cancer, esophagectomy, neoadjuvant chemoradiotherapy tumor positive resection margins

## Abstract

To identify risk factors for tumor positive resection margins after neoadjuvant chemoradiotherapy (nCRT) followed by esophagectomy for esophageal cancer.

**Summary Background Data:**

Esophagectomy after nCRT is associated with tumor positive resection margins in 4% to 9% of patients. This study evaluates potential risk factors for positive resection margins after nCRT followed by esophagectomy.

**Methods:**

All patients who underwent an elective esophagectomy following nCRT in 2011 to 2017 in the Netherlands were included. A multivariable logistic regression was performed to assess the association between potential risk factors and tumor positive resection margins.

**Results:**

In total, 3900 patients were included. Tumor positive resection margins were observed in 150 (4%) patients. Risk factors for tumor positive resection margins included tumor length (in centimeters, OR: 1.1, 95% CI: 1.0–1.1), cT4-stage (OR: 3.0, 95% CI: 1.2–6.7), and an Ivor Lewis esophagectomy (OR: 1.6, 95% CI: 1.0–2.6). Predictors associated with a lower risk of tumor positive resection margins were squamous cell carcinoma (OR: 0.4, 95% CI: 0.2–0.7), distal tumors (OR: 0.5, 95% CI: 0.3–1.0), minimally invasive surgery (OR: 0.6, 95% CI: 0.4–0.9), and a hospital volume of >60 esophagectomies per year (OR: 0.6, 95% CI: 0.4–1.0).

**Conclusions:**

In this nationwide cohort study, tumor and surgical related factors (tumor length, histology, cT-stage, tumor location, surgical procedure, surgical approach, hospital volume) were identified as risk factors for tumor positive resection margins after nCRT for esophageal cancer. These results can be used to improve the radical resection rate by careful selection of patients and surgical approach and are a plea for centralization of esophageal cancer care.

Neoadjuvant chemoradiotherapy (nCRT) before esophagectomy has significantly improved survival of patients with esophageal cancer compared with esophagectomy alone.^1^ One of the objectives of nCRT is to reduce the size of the primary tumor, increasing the probability of a complete resection.^2,3^ However, despite a decrease of 23% that was demonstrated in the CROSS trial, a tumor positive resection margin is still observed in 4.1% to 9% of patients.^1,2,4^ Tumor positive resection margins after esophagectomy are associated with a reduced overall survival caused by local recurrences and distant metastases.^5^

Known risk factors for tumor positive resection margins are mostly derived from patient populations who underwent an upfront esophagectomy in a time before centralization and minimally invasive surgery.^5,6^ In these patients, tumor location, surgical approach (a transhiatal esophagectomy versus a transthoracic esophagectomy) and clinical tumor stage have been identified as risk factors for tumor positive resection margins.^5,7^ However, with both the widespread implementation of nCRT as an integral part of the curative treatment for esophageal cancer and developments in minimally invasive surgery, the influence of these risk factors might have changed in recent years.^1,8,9^

Therefore, the aim of this study was to evaluate the association of patient, tumor, and surgical related factors with tumor positive resection margins in patients treated with nCRT and subsequent esophagectomy for esophageal cancer in a national cohort. To provide insight in hospital volume and the predominant surgical procedures during the study period, these variables were plotted over time.

## Methods

### Study Design

Patient data were collected from the Dutch Upper Gastrointestinal Cancer Audit (DUCA).^10^ The DUCA is a mandatory nationwide quality registry for hospitals performing surgery in patients with gastric and esophageal cancer in the Netherlands. Patient, tumor, and treatment characteristics as well as pathological information and postoperative outcomes (until 30 days after surgery) are registered. This study was approved by the scientific committee of the DUCA and according to the Central Committee on Research involving Human Subjects (CCMO), approval of an ethics committee in the Netherlands was not required.

### Study Population

All patients who underwent an elective esophagectomy after nCRT for esophageal cancer (cT1–4aN0–3M0) between 2011 and 2017 were selected from the DUCA registry. nCRTwas administered according to the CROSS regimen consisting of weekly intravenous administration of carboplatin (area under the curve 2) and paclitaxel (50 mg/m^2^) for 5 weeks with concurrent radiotherapy (41.4 Gy in 23 fractions of 1.8 Gy).^1^ The surgical procedure consisted of either a transhiatal, a McKeown, or an Ivor Lewis procedure. The surgical approach comprised of an open (both abdominal and thoracic phase), a hybrid (minimally invasive thoracic phase or abdominal phase with open thoracic or abdominal phase), or totally minimally invasive esophagectomy (including robot-assisted surgery).

### Primary Outcome and Candidate Predictors

The primary outcome was a tumor positive resection margin which was specified as a macroscopic (R2) or microscopic (R1) tumor positive resection margin according to the American College of Pathologists.^11,12^ As the DUCA registry switched from the definition of tumor positive resection margins according to the Royal College of Pathologists to the definition of the American College of Pathologists in 2014, patients who underwent surgery before 2014 with a tumor positive resection margin between 0.01 mm and 1 mm were redefined as a R0 resection. A tumor positive resection margin could be located either proximal, distal or circumferential.

Candidate predictors for the primary outcome were predefined and based on literature and expert opinion and consisted of age, sex, tumor histology, tumor length (based on endoscopy), tumor location, clinical tumor stage (cT-stage), type of surgery (transhiatal, McKeown esophagectomy, or Ivor Lewis esophagectomy), surgical approach (open, hybrid, or totally minimally invasive), interval between the last day of nCRT and surgery (<6 weeks, 7–12 weeks, >12 weeks), and hospital volume (≤40, 41–60, >60 esophagectomies per year).^5,6,13–16^ Interaction terms of tumor location and surgical procedure as well as hospital volume and surgical approach were added to the multivariable analysis to assess the hypothetical relationship between these candidate predictors. The intended surgical approach was included in the analysis to account for converted procedures.

### Statistical Analysis

Patient and tumor characteristics were described as counts with percentages, means with standard deviations, or medians with ranges where appropriate. The pattern of missing data was analyzed and considered missing at random. As such, missing data was imputed according to the iterative Markov chain Monte Carlo method (with 5 iterations and 5 imputed datasets).^17^ The quantity of missing data per variable before imputation is presented in Table 1. To assess the independent association of the candidate predictors with a tumor positive resection margin, a multivariable logistic regression model was constructed. To identify independent risk factors for tumor positive resection margins, the full model was reduced using backward stepwise elimination according to the Akaike Information Criteria (AIC).^18^ Hence, a candidate predictor was defined as a relevant independent risk factor for the outcome if the predictor was maintained in the final model, regardless of the odds ratio and corresponding 95% confidence interval.^19–21^ The results of the multivariable model were reported as per the TRIPOD guidelines.^20,22^ The AlCs of the modelling steps were included in Supplementary Table 1, http://links.lww.com/ SLA/D300. To provide insight in hospital volume and the predominant surgical procedures during the study period, these variables were plotted over time. Statistical analyses were performed using SPSS Statistics version 25 (IBM Corp., Armonk, NY) and R Studio (Integrated Development Environment for R. RStudio, PBC, Boston, MA). Visual representations were made with GraphPad Prism version 8.0.0 (GraphPad Software, San Diego, CA)

**Table 1:** Patient, Tumor, and Surgical Characteristics of Patients Who Underwent a Curative Esophagectomy After Neoadjuvant Chemoradiotherapy Between 2011 and 2017 in the Netherlands

	No.	%	No.	%	No.	%	
Characteristic	Total		R0		R1		Initial Missing Data (%)
	3900		3750		150		
Sex							0
Male	3041	78	2917	78	124	83	
Female	859	22	833	22	26	17	
Age—mean ± SD	65	± 9.2	65	± 9.2	65	± 8.3	0
BMI— mean ± SD	26	± 4.6	26	± 4.6	26	± 5.3	1
ASA classification							1
1	684	18	667	18	17	11	
2	2395	61	2301	61	94	63	
3	809	21	772	21	37	25	
4	12	0	10	0	2	1	
Histopathology^a^							0
Adenocarcinoma	3077	79	2944	79	133	88	
Squamous cell carcinoma	823	21	806	21	17	12	
Tumor location^b^							0
Proximal esophagus	539	14	519	14	20	13	
Distal esophagus	2600	67	2515	67	85	57	
Gastro-esophageal junction (GEJ)	761	19	716	19	45	30	
Tumor length^a^ (cm)— mean ± SD	5	± 2.9	5	± 2.9	6	± 2.9	14
Clinical T-stage^c^							3
T1	61	2	59	2	2	1	
T2	763	20	743	20	20	13	
T3	2968	75	2849	75	119	80	
T4	108	3	99	3	9	6	
Clinical N-stage							4
N0	1335	34	1299	34	36	25	
N1	1713	44	1641	44	72	48	
N2	740	19	703	19	37	24	
N3	112	3	107	3	5	3	
Surgical procedure—location anastomosis							0
Transhiatal— cervical	1104	28	1060	28	44	29	
Transthoracic— cervical (McKeown)	1332	34	1289	34	43	29	
Transthoracic— thoracic (Ivor Lewis)	1464	38	1401	38	63	42	
Surgical approach							0
Open	1310	34	1246	33	64	43	
Minimally invasive	2425	62	2344	63	81	54	
Hybrid	165	4	160	4	5	3	
Interval end nCRT— surgery							6
< 6 wk	1309	34	1258	34	51	33	
7–12 wk	2271	58	2186	58	85	57	
12 > wk	320	8	306	8	14	10	
Hospital volume							0
< 40	1881	48	1801	48	80	53	
41–60	1123	29	1077	29	46	31	
60>	896	23	872	23	24	16	

a Determined at pretreatment endoscopy.

b Proximal esophagus: cervical, upper one-third and middle one-third of the esophagus, distal esophagus; lower one-third of the esophagus.

c According to the AJCC 7th edition cancer staging manual.

ASA indicates American Society of Anesthesiologists; BMI, preoperative body mass index; cm, centimeter; nCRT, neoadjuvant chemoradiotherapy; R0, tumor-negative resection margin; R1, tumor positive resection margin.

## Results

### Study Population

**Figure 1:**
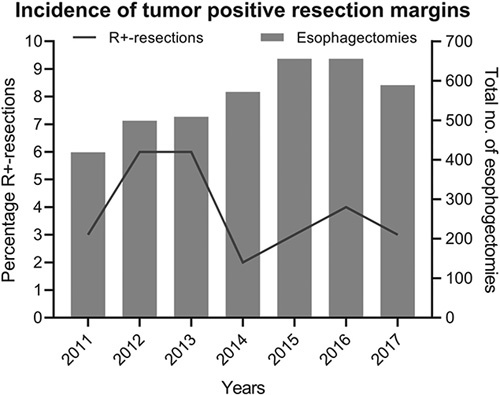
Incidence of tumor positive resection margins (left *y* axis) and the total number of esophagectomies per year (right *y* axis) between 2011 and 2017. R+-resection indicates tumor positive resection margin.

Between 2011 and 2017, a total of 3900 patients who underwent an elective esophagectomy after nCRT for esophageal cancer were eligible for analysis. The majority of patients were male (78%) with a mean age of 65 (± 9.2) years. Most patients had a histologically proven adenocarcinoma (79%) of the distal esophagus (67%) with a clinical T3-stage (75%) and clinically suspected lymph node metastases (66%). An Ivor Lewis esophagectomy was performed in 38% of patients, a McKeown esophagectomy in 34% and a transhiatal esophagectomy in 28%. The majority of the esophagectomies was performed totally minimally invasively (62%). In 2011, 31.5% of the procedures were performed totally minimally invasively, in 2017 this was 79.5%, demonstrating a shift towards totally minimally invasive surgery. A tumor positive resection margin was observed in 150 (4%) patients. As shown in Figure 1, the percentage of tumor positive resection margins between 2011 and 2017 ranged from 2.3% to 5.9%. A detailed overview of patient, tumor, and treatment characteristics stratified for resection status has been provided in Table 1.

### Risk Factors for Tumor Positive Resection Margins

The results of the multivariable logistic regression of the candidate predictors for a tumor positive resection margin are presented in Table 2. Risk factors that were independently associated with an increase in tumor positive resection margins were; tumor length (in centimeters, OR: 1.1, 95% CI: 1.0–1.1), cT4 stage (OR: 3.0, 95% CI: 1.2–6.7, reference: cT1–2 stage), and an Ivor Lewis procedure (OR: 1.6, 95% CI: 1.0–2.6, reference: transhiatal). Squamous cell carcinomas (OR: 0.4, 95% CI: 0.2–0.7, reference: adenocarcinoma), tumors located in the distal esophagus (OR: 0.5, 95% CI: 0.3-1,0, reference: proximal tumors), totally minimally invasive surgery (OR: 0.6, 95% CI: 0.4–0.9, reference: open surgery), and a hospital volume of more than 60 esophagectomies per year (OR: 0.6, 95% CI: 0.4–1.0, reference: ≤40 esophagectomies a year) were associated with a lower risk for tumor positive resection margins. An additional comparison in which the McKeown procedure functioned as reference category was made. This comparison (McKeown (reference), Ivor Lewis, transhiatal) yielded an odds ratio of 1.4 (95% CI: 0.9–2.2) for the Ivor Lewis procedure and of 0.9 (95% CI: 0.5–1.5) for the transhiatal procedure. Candidate predictors that were not independently associated with tumor positive resection margins in the current analysis were: age, sex, and the interval between end of nCRT and surgery. The same holds for the interaction between tumor location and the type of surgical procedure as well as hospital volume and surgical approach.

**Table 2:** Multivariable Logistic Regression Analysis With Positive Resection Margin as Outcome Variable

Risk Factor	Odds Ratio (95% CI)	Regression Coefficient
Histopathology^a^		
Adenocarcinoma	Reference	Reference
Squamous cell carcinoma	0.4 (0.2–0.7)	–0.898
Tumor length (cm)	1.1 (1.0–1.1)	0.083
Tumor location^b^		
Proximal esophagus	Reference	Reference
Distal esophagus	0.5 (0.3–1)	–0.619
Gastro-esophageal junction	0.9 (0.5–1.8)	–0.097
Clinical T-stage^c^		
T1–2	Reference	Reference
T3	1.4 (0.9–2.3)	0.339
T4	3 (1.2–6.7)	1.091
Surgical approach		
Open	Reference	Reference
Minimally invasive	0.6 (0.4–0.9)	–0.514
Hybrid	0.5 (0.2–1.2)	–0.655
Type of surgery—location anastomosis		
Transhiatal—cervical	Reference	Reference
Transthoracic—cervical (McKeown)	1.2 (0.7–1.9)	0.127
Transthoracic—thoracic (Ivor Lewis)	1.6 (1.0–2.6)	0.489
Hospital volume		
≤40	Reference	Reference
41–60	1 (0.7–1.5)	0.039
60>	0.6 (0.4–1)	–0.443

a Determined at pretreatment endoscopy.

b Proximal esophagus: cervical, upper one-third and middle one-third of the esophagus, distal esophagus; lower one-third of the esophagus.

c According to the AJCC 7th edition cancer staging manual.

95% CI indicates 95% confidence interval; BMI, preoperative body mass index; cm, centimeter; nCRT, neoadjuvant chemoradiotherapy.

### Hospital Volume and Surgical Trends Over Time

**Figure 2:**
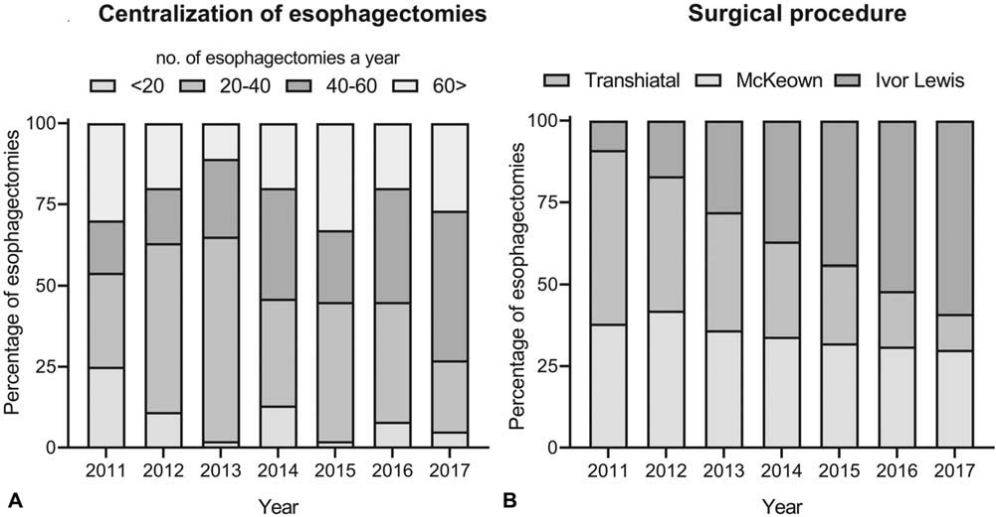
A, Centralization of esophagectomies toward larger volume centers between 2011 and 2017. B, Trends in type of esophagectomy between 2011 and 2017.

Between 2011 and 2013, the majority of patients underwent surgery in hospitals that performed <40 esophagectomies a year. In 2012, a minimum of 20 esophagectomies a year was implemented as a quality measure for hospitals performing esophagectomies. As a result, an increasing number of patients underwent surgery in hospitals performing 20 to 40 esophagectomies a year. After 2013, the majority of patients (54%) underwent surgery in hospitals performing >40 esophagectomies a year. The centralization of esophagectomies in the Netherlands is shown in Figure 2A.

Between 2011 and 2014, the transhiatal and McKeown esophagectomy were the predominant procedures that were performed. After 2014, the Ivor Lewis esophagectomy was the most frequently performed surgical procedure. The time trends regarding these surgical procedures are shown in Figure 2B.

**Figure 3:**
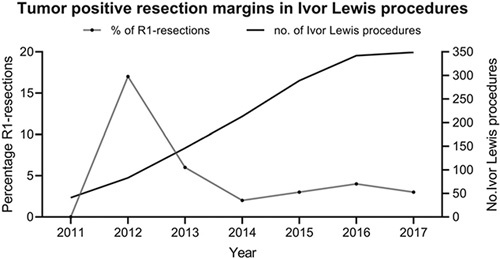
The number of positive resection margins in Ivor Lewis procedures (left y axis) relative to the total number of Ivor Lewis procedures (right y axis) between 2011 and 2017. R1-resection indicates tumor positive resection margin.

During the introduction of the Ivor Lewis procedure in 2012 the incidence of tumor positive resection margins was 17%. After 2012, with an increase in the number of Ivor Lewis procedures over the years, a decrease in the percentage of tumor positive resection margins during this procedure was observed. The time trends for the percentage of tumor positive resection margins during an Ivor Lewis procedure and the number of Ivor Lewis procedures per year are shown in Figure 3.

## Discussion

This national multicenter cohort study demonstrates that patients with larger tumors (reflected by tumor length and clinical tumor stage) and patients who undergo an Ivor Lewis procedure have an increased risk of tumor positive resection margins after nCRT. In contrast, squamous cell carcinomas, tumors located in the distal esophagus, minimally invasive surgery, and ahospital volume of >60 esophagectomies per year were associated with a decreased risk of tumor positive resection margins. This information could aid in selecting the right surgical approach and provides opportunities to improve esophageal cancer care.

The present study adds to the current literature on favorable postoperative results in high-volume centers.^16,23–28^ Our results suggest that to reduce the occurrence of an incomplete resection after neoadjuvant treatment the annual hospital volume should be at least over 40, but preferably, over 60 resections a year. This result is in line with previous literature assessing survival in esophagectomies and provides a valid argument to centralize the highly specialized care of esophageal cancer patients.^16^ Additionally, recent literature stated that an annual hospital volume of over 10 esophagectomies a year was already associated with a reduced occurrence of tumor positive resection margins.^6^ However, the results of the current study suggest that, in the setting of a mandatory hospital volume of at least 20 esophagectomies a year, as in the Netherlands, radical resections rates keep improving with further centralization.^23–28^

In contrast to previous studies, the current study demonstrates a reduced risk of tumor positive resection margins after minimally invasive surgery. A previous report demonstrated an increase in tumor positive resection margins after laparoscopic surgery and claimed this was due to inexperience of the surgeons.^6^ In the report of *Schlick et al.* the mean annual hospital volume was considerably lower than the obliged esophagectomy rate of 20 per year in the Netherlands. This could be explained by the reduced risk of tumor positive resection margins after minimally invasive surgery that was found in the current analysis. It might well be hypothesized that the benefits of minimally invasive surgery only emerge in a cohort in which the overall hospital volume is relatively high as in the present cohort. A high hospital volume allows surgeons to rapidly complete the learning curve that is associated with minimally invasive esophagectomies as well as maintaining the routine of this procedure resulting in fewer incomplete resections.^29,30^

The demonstrated increased probability of tumor positive resection margins in an Ivor Lewis procedure compared to a transhiatal esophagectomy might be attributable to a learning curve and a shift in the surgical landscape toward minimally invasive surgery.^30^ The Ivor Lewis approach was introduced during the beginning of the study period and has become the predominant approach over the years in the Netherlands. Together with the introduction of minimally invasive surgery this has led, initially, to an increase in tumor positive resection margins in Ivor Lewis procedures. This increase was fortunately followed by a rapid decrease in positive resection margins which suggest the presence of a learning curve (as demonstrated in Figure 2). The increased probability of tumor positive resection margin following an Ivor Lewis esophagectomy could be related to poor patient selection and choice of surgical approach during the initial learning phase of this technique. The indication for a tran-shiatal procedure for distal tumors or a McKeown procedure for middle and proximal tumors has been well defined. On the other hand, the Ivor Lewis procedure was initially introduced as a procedure for tumors located in the middle and distal esophagus. However, over the years it has become increasingly clear that the Ivor Lewis procedure is best suited for tumors of the distal esophagus.^15,31,32^ During the introduction period of this procedure, it is possible that a number of patients underwent an Ivor Lewis procedure in whom a McKeown was more appropriate. This might have led to the relatively high number of tumor positive resection margins after Ivor Lewis procedures in this period.

This is the first study to assess risk factors for tumor positive resection margins in a patient population that in its entirety was treated according to the same nCRT regimen. It was hypothesized that the effects of risk factors such as tumor length and cT-stage would have been of lesser influence since downstaging is one of the primary aims of nCRT. Our results demonstrate that even after nCRT, larger and more invasive tumors (reflected by tumor length and cT-stage) before treatment remain risk factors for an incomplete resection. This might be explained by previous literature which demonstrated that the relative tumor regression (ie, downstaging) during nCRT is comparable between larger and smaller tumors.^3^ Furthermore, it is well known that squamous cell carcinomas tend to respond better to nCRT than adenocarcinomas which leads to a higher percentage of pathologic complete responders in this group.^1^ Presumably, this has led to the reduced risk for tumor positive resection margins in squamous cell carcinomas that is observed in the current report. Since histopathology is not a modifiable risk factor it is hard to practically apply this finding. Nevertheless, the favorable outcome that is associated with squamous cell carcinomas can be used during the clinical decision-making of treatment in patients with esophageal cancer.

The interval between the completion of nCRT and surgery was not identified as a risk factor for tumor positive resection margins in the current report. Based on previous literature, it was hypothesized that after a short interval (ie, <6 weeks) the effects of nCRT, such as inflammation and volume regression, would still be in effect increasing the probability of positive resection margins. For larger intervals (ie, >12 weeks) it was hypothesized that radiation effects such as fibrosis would lead to more complex surgical procedures and therefore to more tumor positive margins.^33^ The present analysis implies that even though the aforementioned processes might take place they do not result in a higher incidence of tumor positive resection margins.

To further reduce the incidence of tumor positive resection margins and to aid clinical decision-making with regard to patients who might not benefit from an esophagectomy after nCRT, the assessment of resectability during restaging can be improved. Currently, the main goal of restaging is to rule out the presence of interval distant metastases after nCRT by positron emission tomography -computed tomography.^34^ Detailed locoregional assessment of the primary tumor might identify patients in whom a radical resection is challenging. Currently, conventional imaging modalities such as contrast enhanced CT do not provide sufficient detail to accurately assess tumor invasion and therefore resectability.^35^ Modalities such as magnetic resonance imaging which provide superior soft tissue contrast might be used in the assessment of resectability and further increase the radical resection rate.^36,37^

The use of prospectively collected data from a national registry with a marginal amount of missing data and the homogeneity of the study population in terms of neoadjuvant treatment were considered significant strengths of the current study. However, some detailed information was not collected in the registry. For the current report it would have been informative to analyze the location of the tumor positive resection margin (ie, circumferential, proximal, and distal) since this might provide insight as to whether the proper surgical procedure was performed regarding the location of the tumor. Nevertheless, we believed that the provided dataset was of sufficient quality to answer our research question. The use of population based data could be considered a limitation of the current study, as it could introduce selection bias. As such, it is important to note that the identified risk factors should be considered risk factors for the outcome and are not causally related to the outcome. Furthermore, results from large population-based studies are extremely valuable, as they are based on and therefore generalizable to patients who are encountered in daily clinical practice.

In conclusion, this report identified the risk factors for tumor positive resection margins after esophagectomy in patients who underwent nCRT and provides insights on how to reduce the occurrence of this unfavorable prognostic factor. Additionally, current results confirm the safety of a minimally invasive esophagectomy after nCRT and provide an incentive to persevere with the centralization of esophageal cancer care.

## Supplementary Material
