# Supplementary material for: Risk Factors for Tumor Positive Resection Margins After Neoadjuvant Chemoradiotherapy for Esophageal Cancer: Results From the Dutch Upper GI Cancer Audit: A Nationwide Population-Based Study
Source: Ann Surg. 2023 Jan 10;277(2):e313–9. doi: 10.1097/SLA.0000000000005112 (PMC9831046; doi:10.1097/SLA.0000000000005112)
Supplement: Supplementary file 1 [file sla-277-e313-s001.docx]

| **A. Single term deletions of full model with interaction** | **AIC** |
| --- | --- |
| None | 50.000 |
| Sex | 48.420 |
| Age | 49.172 |
| Histopathology | 56.438 |
| Tumor length | 57.085 |
| cT-stage | 51.587 |
| Interval end nCRT - surgery | 46.826 |
| Interaction: tumor location*Type of surgery | 43.058 |
| Interaction: hospital volume*Surgical approach | 47.641 |
|  |  |
| **B. Single term deletions of full model** | **AIC** |
| Full model | 34.000 |
| Sex | 32.417 |
| Age | 33.318 |
| Histopathology | 40.805 |
| Tumor length | 40.812 |
| Tumor location | 39.783 |
| cT-stage | 36.054 |
| Type of surgery | 35.454 |
| Surgical approach | 37.260 |
| Hospital volume | 34.486 |
| Interval end nCRT - surgery | 30.603 |
|  |  |
| **C. Single term deletion of final model** | **AIC** |
| Final model | 26.000 |
| Histopathology | 33.810 |
| Tumor length | 32.788 |
| Tumor location | 31.595 |
| cT-stage | 28.063 |
| Type of surgery | 27.165 |
| Surgical approach | 28.652 |
| Hospital volume  **A.** Assessment of interaction terms. Interaction terms were excluded based on the decrease in the Akaike Information Criteria (AIC). **B.** AIC of the full model and the effect of deleting individual candidate predictors. **C.** AIC of the final model and the effect of deleting individual predictors. | 26.346 |

**Supplementary 1.** Model development
